# LIR-1 and PDTF-1 regulate the permeability barrier function of the *C. elegans* cuticle

**DOI:** 10.17912/micropub.biology.000434

**Published:** 2021-07-27

**Authors:** Anjali Sandhu, Riya Sheokand, Varsha Singh

**Affiliations:** 1 Department of Molecular Reproduction, Development and Genetics, Indian Institute of Science, Bangalore, India 560012

## Abstract

The cuticle of *C. elegans* presents the second largest surface area in the soma of worms. We have recently reported that the permeability barrier in the cuticle is dependent on six permeability-determining (PD) collagens and BLMP-1, a transcription factor. To identify additional regulators of cuticle permeability, we performed RNA interference for 286 transcription factors expressed in the *C. elegans* hypodermis and studied cuticle permeability to Hoechst 33258, a nucleic acid dye. Although the cuticle of wild type N2 strain is impermeable to this dye, RNAi of *lir-1* or T26A8.1 (PDTF-1) caused permeability defect in the cuticle. LIR- 1 and PDTF-1 positively regulate expression of collagen and/or collagen processing enzymes. As a consequence, *lir-1* and *pdtf-1* RNAi also caused enhanced susceptibility to exogenous toxins such as paraquat, levamisole and ivermectin. Thus, LIR-1 and PDTF-1 are two hypodermis-specific transcriptional regulators of cuticle permeability.

**Figure 1. Transcription factors LIR-1 and PDTF-1 regulate permeability of C. elegans cuticle.**
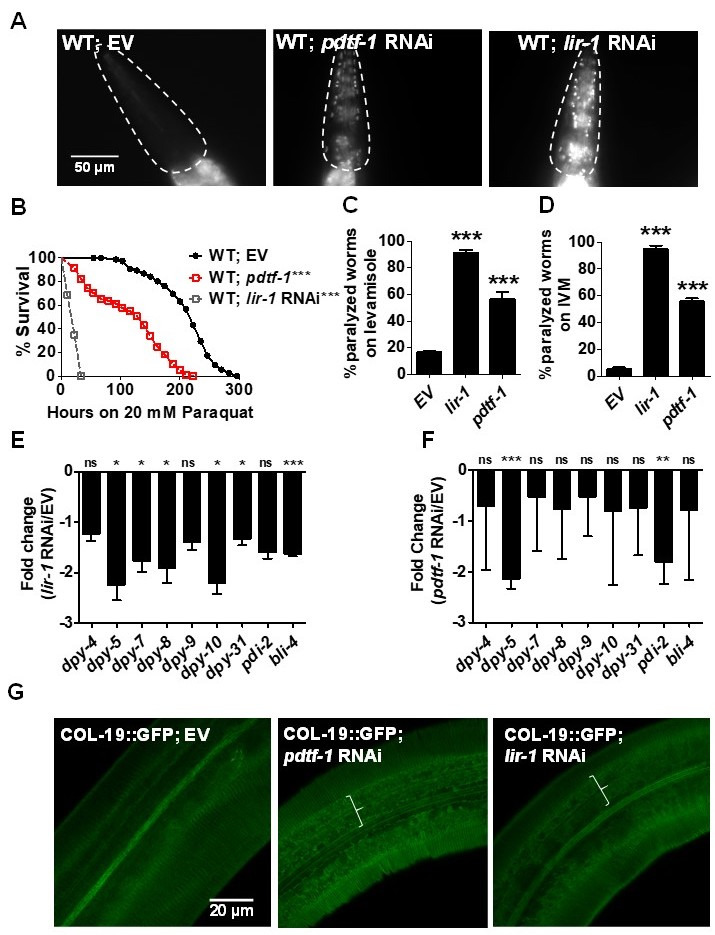
Hoechst 33258 permeability assay in (A) empty vector control (EV), *pdtf-1* (T26A8.1), and *lir-1* RNAi in WT animals. Dashed line outlines the pharynx. Scale bar, 50 μm. n=3; N≥15. Kaplan Meier survival curves of (B) EV control, *pdtf-1,* and *lir-1* RNAi animals against 20 mM PQ. n=3; N≥50. Percent paralyzed of EV *, lir-1,* and *pdtf-1* RNAi in WT animals upon (C) 30 minutes exposure to 125 μM levamisole and, (D) 8 minutes exposure to 50 μM Ivermectin (IVM). n=3; N≥30. Real time PCR analysis of transcripts for collagens and collagen processing enzymes upon (E) *lir-1* RNAi and (F) *pdtf-1* RNAi *in* WT animals compared to EV control. P *col-19* ::GFP expression in (G) EV, *pdtf-1* , and *lir-1* RNAi animals. Areas with disruption of annuli and furrows are marked. Error bars indicates SEM. *, *p≤* 0.05; **, *p≤* 0.005; ***, *p≤* 0.0005; ns-not significant, significance based on Student’s *t* test and Mantel Cox test for histograms and survival curves respectively. *p* value for survival curves are indicated next to genotypes.

## Description

*C. elegans* skin consists of two layers- cuticle and hypodermis. The cuticle is the outer most layer that is predominately composed of cross-linked collagens. It acts as a physical barrier against exogenous toxins and helps in locomotion (Page and Johnstone 2007; Altun and Hall 2009; Sandhu *et al.* 2021). Cuticle is secreted by the underlying hypodermis, a single cell layer. In a recent study, we uncovered the role of six collagens- DPY-2, -3, -7, -8, -9 and -10 in maintenance of permeability barrier function of the cuticle to a nucleic acid dye Hoechst 33258. Lack of any of these collagens or transcription factor BLMP-1 led to enhanced Hoechst staining and susceptibility to exogenous toxins (Bus *et al.* 1976; Kass *et al.* 1980; Bus and Gibson 1984; Atchison *et al.* 1992; Sandhu *et al.* 2021). To see if there were additional regulators of cuticle permeability, we performed an RNAi screen for 286 transcription factors known to be expressed in the hypodermis (Kaletsky *et al.* 2018) using Hoechst 33258 staining assay (Extended Data, Table 1). Animals treated with empty vector RNAi control remained impermeable to Hoechst stain. As expected, we found that *blmp-1* RNAi caused staining (Extended Data, Table 1; Sandhu *et al.* 2020). In addition, we found that two transcription factors, *lir-1* and T26A8.4, also caused staining (Fig. 1A). We name T26A8.4 permeability-determining transcription factor PDTF-1. Importantly, RNAi of either *lir-1* or *pdtf-1* in WT animals enhanced their susceptibility towards exogenous toxins such as paraquat (PQ), levamisole, and ivermectin (IVM) (Fig. 1, B-D). This could result from their ability to regulate collagen expression or through other mechanism(s). To test the former, we examined the effect of *lir-1* and *pdtf-1* RNAi on COL-19::GFP expression, reporter for adult collagen COL-19 (Thein *et al.* 2003) expressed in circumferential ridges called annuli in *C. elegans* cuticle. Furrows delineate the annuli which pattern the entire *C. elegans* cuticle. COL-19::GFP showed regular expression in annuli separated by parallel furrows in wild type animals as expected, whereas its expression was disrupted proximal to alae in both *lir-1* and *pdtf-1* RNAi animals (marked in Fig. 1G). To test if permeability determining transcription factors regulate PD collagen expression, we examined level of transcripts for collagens and collagen processing enzymes in *lir-1* and *pdtf-1* RNAi animals by qRT-PCR (Fig. 1, E-F). We found that expression of transcripts for PD collagens as well as DPY-4 and DPY-5 *collagens* was reduced in *lir-1* RNAi animals. *pdtf-1* RNAi only altered the expression of transcripts for DPY-5 collagen and PDI-2, a collagen processing enzyme. This is consistent with our previous study where we showed that *pdi-2* knockdown also causes permeability defects (Sandhu *et al.* 2021). In all, our analysis of transcription factors in the hypodermis indicates that LIR-1 and PDTF-1 are transcription regulators of cuticle permeability barrier function, collagen expression, and protection of *C. elegans* from exogenous toxins.

## Methods

**Strains used in the study**

*C. elegans* strains used in the study were wild-type N2 (Bristol) and TP12 (P *col-19* ::GFP).

**RNAi interference**

Systemic RNA interference was done as described (Fraser *et al.* 2000; Kamath *et al.* 2001). HT115(DE3), an *E. coli* strain, expressing double-stranded RNA for the target gene was grown in LB broth containing carbenicillin (50 g/ml), 8 hours, at 37°C. Bacterial culture was plated onto NGM plates containing 50 g/ml carbenicillin and 5 mM isopropyl D-thiogalactoside (IPTG) and incubated at 25°C for 12 hours before use. Clones were confirmed by sequencing. Gravid adults were allowed to lay eggs on empty vector control or target RNAi plates. Eggs were allowed to grow at 20°C until L4 stage for RT PCR analysis or until the young adult stage for survival and Hoechst staining experiments.

**Cuticle permeability assay**

Cuticle permeability was assessed using Hoechst 33258 stain animals as described (Moribe *et al.* 2004). Gravid adults were allowed to lay eggs on empty vector control or target RNAi plates. Eggs were allowed to grow at 20°C until young adult stage. Worms were stained with 10 ug/ml Hoechst 33258 for 30 mins at room temperature (RT). Unbound stain was removed by washing with M9 buffer before visualization and imaging under DAPI filter using Zeiss Apotome.

**Quantitative Real-time PCR**

Synchronized L4 nematodes grown on control or target RNAi at 20°C were harvested by washing the plates with M9 buffer and frozen in TRizol reagent at -80°C. RNA was extracted using RNeasy Plus Universal Mini Kit according to the manufacturer’s instruction (Qiagen). cDNA was prepared using the iScript cDNA synthesis kit (BIO-RAD). qRT-PCR was conducted using the BIO-RAD TaqMan One-Step Real-time PCR protocol using SYBR Green fluorescence (BIORAD) on an Applied Biosystems QuantStudio 3 real-time PCR machine in 96-well plate format. Fifty nanograms of RNA were used for real-time PCR. 10 ul reactions were set-up in two replicates. Relative -fold changes were calculated using the comparative *CT (2 -ΔΔCT* ) method and normalized to actin-1 (Livak and Schmittgen 2001). Three or more biological replicates were used for qRT-PCR analysis.

**Survival assay**

Young adult animals were synchronized on control or target RNAi bacterial plates at 20°C. ≥50 animals were exposed to 20 mM paraquat on NGM plates with OP50 at 20°C for oxidative stress induction. Animals were scored for survival every 6-8 hours (Park *et al.* 2009; Sandhu *et al.* 2021).

**Paralysis assay**

For paralysis experiments, animals were synchronized on control or target RNAi plates until young adult stage at 20°C. Levamisole assays were done by scoring the number of paralyzed worms after a 30 mins exposure to 125 μM levamisole on NGM plates with OP50 at RT (Lewis *et al.* 1980). For ivermectin stress, adult animals were exposed to 50 μM ivermectin in M9 buffer and scored for paralysis at 8 minutes at RT (Kass *et al.* 1980).

**Statistical analysis**

Survival assays were plotted using the PRISM 5.01 (Kaplan-Meier method). Survival curves with *p* values <0.05 for Mental-Cox test were considered significantly different. Statistics for survival assays are presented as extended data in Table 2.

**Extended Data**

Table 1. List of 286 Transcription factors RNAi clones tested for Hoechst 33258 permeability. https://doi.org/10.22002/D1.2053

Table 2. Statistics for survival assays. https://doi.org/10.22002/D1.2054

## Reagents

Reagents used for the study were paraquat (methyl viologen dichloride hydrate) (Sigma, cat#856177), levamisole (Sigma, cat# 1916142), ivermectin (Sigma, cat# 18898), and Hoechst 33258 (Sigma, cat#94403).
